# Multisystem Complications in Postpartum-Onset Evans Syndrome: A Case Report

**DOI:** 10.7759/cureus.100196

**Published:** 2025-12-27

**Authors:** Kiya Shazadeh Safavi, Kelly Garcia Chavez, Faisal Shariff, Faiza Fakhar, Nezam Altorok

**Affiliations:** 1 Internal Medicine, The University of Toledo College of Medicine and Life Sciences, Toledo, USA; 2 College of Nursing, The University of Toledo, Toledo, USA

**Keywords:** acute hemolytic anemia, autoimmune hemolytic anemia (aiha), evans syndrome, immune thrombocytopenia (itp), treatment for evans syndrome

## Abstract

A 35-year-old postpartum woman presented with gastrointestinal symptoms, anemia, thrombocytopenia, and acute kidney injury (AKI) and later suffered from splenic rupture. After extensive evaluation, she was diagnosed with Evans syndrome (ES). ES is characterized by the co-occurrence of multiple autoimmune cytopenias (AIC), most commonly autoimmune hemolytic anemias (AIHA) and immune thrombocytopenia (ITP). Since the incidence is exceedingly rare, the management is considerably difficult due to a lack of structured guidelines, leading to considerable patient morbidity. Our patient’s treatment regimen included combinations of steroids, IV immunoglobulin, mycophenolate, and eculizumab. Here, we highlight the diagnostic complexity and therapeutic challenges associated with ES, particularly when triggered by pregnancy or infection.

## Introduction

Evans syndrome (ES) is a rare disease characterized by the co-occurrence of autoimmune cytopenias (AIC), most often autoimmune hemolytic anemia (AIHA) and immune thrombocytopenia (ITP) and/or immune neutropenia [1]. Typical presentations include anemia, thrombocytopenia, jaundice, and splenomegaly. As of 2016, the incidence and prevalence of ES are 1.8 per million persons per year and 21.3 per million persons, respectively, with a median survival of 7.2 years [2]. Due to the rarity of ES, treatment recommendations are largely extrapolated from those of isolated AIC. Furthermore, treatment is considered more difficult when compared with isolated warm AIHA, as evidenced by lower responses to standard therapy, more frequent relapses, and higher mortality. Initial therapy typically includes a combination of glucocorticoids, rituximab, and mycophenolate mofetil. Case reports describe refractory disease despite additional IV immunoglobulin and splenectomy and discuss the utility of allogenic hematopoietic stem cell transplantation; however, data are largely limited given the rarity of the disease [3-5]. A case series with 68 patients, all treated with corticosteroids, 50 of whom required second-line treatment, documented short-term response in approximately 80%; however, at median follow-up (4.8 years), only 32% were in remission, and 24% had passed [6]. Despite advances in treatment, recent studies have demonstrated higher mortality associated with ES when compared to isolated AIC, supporting the need for further refinement of ES management [7]. Therefore, we present a case of an otherwise healthy 35-year-old postpartum female with equivocal lab findings and genetic testing who was found to have treatment-resistant ES.

## Case presentation

A seven-month postpartum female in her mid-30s presented with malaise, nausea, vomiting, and diarrhea. Notable history included a delayed postpartum hemorrhage one week after delivery, requiring dilation and curettage. Her laboratory evaluation was notable for an acute kidney injury (AKI) with creatinine (Cr) of 2.2 mg/dL, normocytic anemia (hemoglobin 8.4 g/dL), thrombocytopenia (platelet 30k/μL), elevated lactate dehydrogenase (2,150 IU/L) and D-dimer (630 ng/mL), low haptoglobin (<30 mg/dL) with an elevated reticulocyte count (3.4%), suggestive of hemolysis, and a positive fecal occult blood test, suggesting some amount of gastrointestinal bleeding (Table 1).

**Table 1:** Patient lab values with reference ranges.

Lab Test	Patient Value	Reference Range
Creatinine	2.2 mg/dL	0.4 – 1.0 mg/dL
Hemoglobin (Initial, Later)	8.4 g/dL, 10.5 g/dL	12 – 16 g/dL (female)
Platelet Count (Initial, Later)	30,000 /μL, 38,000 /μL	150,000 – 450,000 /μL
Lactate Dehydrogenase (LDH)	2,150 IU/L	100 – 235 IU/L
D-dimer	630 ng/mL	< 255 ng/mL
Haptoglobin	<30 mg/dL	32 – 228 mg/dL
Reticulocyte Count	3.40%	0.4 – 2.2%
C3 (Complement)	57 mg/dL (initial), 70 mg/dL (repeat)	75 – 175 mg/dL
C4 (Complement)	Normal	14 – 40 mg/dL
ADAMTS13 Activity	Mildly reduced ~60% (initial), 66% (later)	>67% (usually), severe deficiency <10%
Alternative Pathway Activity	<10%	>46%
Factor H Level	16.5 mg/dL	18.5 – 40.8 mg/dL
Factor H Autoantibodies	<4.0 U/mL (equivocal)	<15.8 U/mL

Later that day, she developed sudden abdominal pain and hemorrhagic shock secondary to splenic rupture, necessitating splenic artery embolization, massive transfusion, and dialysis. A blood smear revealed schistocytes and occasional microspherocytes, but a direct Coombs test (DAT) was negative, suggesting possible DAT-negative warm AIHA. The infectious workup, including hepatitis, Epstein-Barr virus (EBV), and antinuclear antibody/antinuclear cytoplasmic antibody, was negative. Complement testing showed low C3 (57 mg/dL) with normal C4. ADAMTS13 activity was mildly reduced without a detectable inhibitor, arguing against thrombotic thrombocytopenic purpura (TTP). Positive cytomegalovirus (CMV) IgM/IgG serologies and PCR confirmed viremia (1050 IU/mL), raising concern for CMV-induced, complement-mediated, thrombotic microangiopathy (TMA), specifically atypical hemolytic uremic syndrome (aHUS) secondary to CMV. Aside from the recent pregnancy, there was no clear explanation as to how this developed, given her immunocompetence; however, her children in day care were the presumed source. Initial treatment included brief plasmapheresis (discontinued following a negative ADAMTS13 inhibitor), a five-week prednisone taper, and a three-week valganciclovir course. Her hemoglobin and platelets normalized, though CMV viral load remained mildly elevated at follow-up.

Four months later, she presented with fatigue, upper respiratory symptoms, and dark urine. Labs showed mild anemia (Hb 10.5 g/dL), thrombocytopenia (platelet 38K/μL), indirect hyperbilirubinemia, and worsening anemia. She tested positive for COVID-19 but was CMV/EBV DNA-negative. ADAMTS13 activity was 66%, and no schistocytes were seen on peripheral smear. Bone marrow biopsy revealed relative erythroid and megakaryocytic hyperplasia and increased iron stores, consistent with reactive marrow. Repeat complement levels showed low C3. Given clinical suspicion of recurrent TMA, prednisone was started. The repeat DAT returned weakly positive for IgG, consistent with warm AIHA. The combination of warm AIHA and ITP yielded a diagnosis of ES. Complement pathway genetic testing showed heterozygous risk factors/modifiers in genes encoding complement factor H (CFH, a glycoprotein regulating the alternate complement pathway) and thrombomodulin. Regarding CFH, she was heterozygous for CFH c.163G>A, p.Gly55Arg. The wild-type glycine was noted as highly conserved between species. This missense variant occurred in a residue predicted to impact protein function and possible associations with autosomal dominant or recessive CFH deficiency and increased susceptibility to aHUS. However, this variant was not reported in large databases and not seen in other affected individuals and was thus labelled as a variant of undetermined significance. Furthermore, she was heterozygous for large deletions in the CFHR1 (exons 2-6) and CFHR3 (exons 1-6) gene clusters. Since this cluster is prone to structural variation, the clinical significance was again uncertain. There was another missense variation in the thrombomodulin-encoding THBD gene (THBD c.1601C>T, p.Ala534Val). Preliminary evidence showed this gene may be involved in C3 glomerulopathy and susceptibility to aHUS; however, it is unclear if this variation altered protein function. Alternative complement pathway testing showed dysregulation with lowered function (<10%, normal >46%), normal C4, low C3 (70 mg/dL, normal 75-175 mg/dL), and low factor H (16.5 mg/dL, normal 18.5-40.8 mg/dL). Factor H autoantibody testing was equivocal (<4.0 U/mL, normal <15.8). 

Her recurrent aHUS was presumed secondary to factor H deficiency; however, it was unclear if it was autoantibody-mediated. Regardless, her treatment course has been complex. She initially received multiple glucocorticoid regimens. Given the suboptimal response, she was placed on a four-week rituxan regimen. She had another admission for aHUS and was treated with IV immunoglobulin, glucocorticoids, and mycophenolate 1000 mg twice daily, which she responded to well. Afterwards, mycophenolate 500 mg daily was continued for chronic management. It was intermittently held due to elevated liver function testing or viral symptoms, but otherwise tolerated well for nearly two years. Later, she had yet another episode of aHUS with anemia, thrombocytopenia, and AKI, requiring IV immunoglobulin and glucocorticoids, and was started on IV eculizumab (induction: 900 mg weekly for four doses; maintenance: 1.2 g at week 5, then 1.2 g every two weeks thereafter) for chronic management with a good response. She follows up with hematology monthly with an interval complete blood count (CBC), comprehensive metabolic panel (CMP), and hemolysis labs, and it has been noted that she has tolerated the eculizumab well for approximately nine months now.

## Discussion

This case underscores the diagnostic complexity and unpredictable course of ES in adults, particularly in the context of pregnancy-associated immune shifts and infectious triggers. Splenic rupture may have been an early manifestation of severe thrombocytopenia or underlying vascular fragility in the context of complement dysregulation. The subsequent evolution of a positive DAT emphasizes the dynamic immunohematologic profile in ES, necessitating serial assessments.

In a multi-center study of 68 adult ES patients, 80% responded to corticosteroids initially, but long-term remission was achieved in only 32% at a median follow-up of 4.8 years [6]. Our patient's clinical trajectory reflects this, with partial responses to first-line therapy and relapse necessitating multi-agent immunosuppression. As with other reports, the use of rituximab and mycophenolate reflects an extrapolation from AIHA and ITP treatment protocols due to the lack of ES-specific guidelines [3-5,8]. Regarding refractory disease, some authors have noted the utility of danazol and cyclosporin A as salvage treatment [9,10]. While allogeneic hematopoietic stem cell transplantation has been explored for refractory disease, data remain sparse and are largely limited to pediatric or case-report-level evidence [5].

Regarding our patient, the precipitating factors that surround her initial presentation remain unclear. Although the exact mechanism is unknown, some have shown an increased incidence and disease progression surrounding the peripartum state. This is thought to be contributed to pregnancy-induced immune dysregulation and physiologic complement abnormalities surrounding pregnancy [7]. 

This case illustrates the importance of considering ES in adults presenting with relapsing cytopenias, especially when initial autoimmune markers are equivocal. It reinforces the necessity of repeat diagnostic evaluation, given the potential for evolving serologies and bone marrow findings. Finally, the mortality associated with ES remains significantly higher than isolated AIHA or ITP, reinforcing the need for an individualized approach and long-term monitoring [2,7].

## Conclusions

The diagnosis of ES can be challenging due to its exceedingly rare occurrence and diagnostic ambiguity, especially in early stages when autoimmune markers like DAT may be negative. Serial evaluations are critical for accurate diagnosis. This case highlights the utility of genetic testing in atypical presentations of ES with high clinical suspicion and equivocal lab findings. Furthermore, management of refractory ES requires a tailored multi-agent approach, often involving immunosuppressants and complement inhibitors like eculizumab. Due to a lack of standardized treatment guidelines, high relapse rates, and significant morbidity, close monitoring by a hematologist may be beneficial.
